# Bird Integumentary Melanins: Biosynthesis, Forms, Function and Evolution

**DOI:** 10.3390/ijms17040520

**Published:** 2016-04-08

**Authors:** Ismael Galván, Francisco Solano

**Affiliations:** 1 Department of Evolutionary Ecology, Doñana Biological Station—CSIC, 41092 Sevilla, Spain; galvan@ebd.csic.es; 2 Department of Biochemistry and Molecular Biology B & Immunology, School of Medicine and IMIB, University of Murcia, 30100 Murcia, Spain

**Keywords:** melanogenesis, pheomelanin, eumelanin, avian melanins

## Abstract

Melanins are the ubiquitous pigments distributed in nature. They are one of the main pigments responsible for colors in living cells. Birds are among the most diverse animals regarding melanin-based coloration, especially in the plumage, although they also pigment bare parts of the integument. This review is devoted to the main characteristics of bird melanins, including updated views of the formation and nature of melanin granules, whose interest has been raised in the last years for inferring the color of extinct birds and non-avian theropod dinosaurs using resistant fossil feathers. The molecular structure of the two main types of melanin, eumelanin and pheomelanin, and the environmental and genetic factors that regulate avian melanogenesis are also presented, establishing the main relationship between them. Finally, the special functions of melanin in bird feathers are also discussed, emphasizing the aspects more closely related to these animals, such as honest signaling, and the factors that may drive the evolution of pheomelanin and pheomelanin-based color traits, an issue for which birds have been pioneer study models.

## 1. Introduction

Color is one of the traits that most conditions the appearance of animals. Animal coloration is extraordinarily diverse, and this diversity attracted the attention of early scientists and it eventually played an important role in the development of paradigms in biology such as the speciation theory [1]. Indeed, the first attempts to explain the origin of this diversity in colors precede modern studies on evolution and adaptation [2]. Color largely affects the way animals communicate with others by fulfilling functions such as concealment and signaling of genotypic quality to conspecifics, and coloration plays a relevant role in the evolution of animal life histories [3].

Animal coloration is the result of two different but related physical processes: (a) the direct absorption of specific wavelengths of light by natural pigments, which could be termed pigmentary colors; and (b) the interference of light reflected by biological microstructures with contrasting refractive indices, which creates the so-called structural colors. The latter mechanism allows producing colors that cannot be generated by pigments alone, but specialized microstructures often require the presence of pigments that absorb certain wavelengths to produce structural colors [4]. Therefore, pigmentary and structural colors are not the result of two independent processes, but rather pigments are the main basis responsible for the entire diversity of animal coloration.

Among animals, the colors of birds are particularly diverse, which is related to the fact that, contrary to other vertebrates such as mammals but in common to humans, birds mainly rely on visual cues for communication [5]. The pigments responsible for this diversity are deposited in feathers and bare parts such as bills and legs. These pigments are, in alphabetical order: carotenoids, flavins, melanins, porphyrins, psittacofulvins, pterins, purines and turacin. Most of these pigments are present in only certain groups of birds. Melanins and carotenoids are widespread among birds and therefore represent the main contribution mechanisms to avian color diversity, but usually melanins are more abundant than carotenoids and in some species, such as barn swallows, the levels of melanin are orders of magnitude greater than carotenoids [6,7,8].

Melanin is the generic name used to refer to perhaps the most ubiquitous, resistant, heterogeneous and ancient polymer found in nature [9]. As in mammalian skin and hair, the integument of birds contains two chemical forms of melanin: eumelanin, which gives rise to dark black, brown or grey colorations, and pheomelanin, which gives rise to lighter yellowish to reddish colorations. Here, we aim at reviewing the mechanisms by which birds produce melanins and melanin-based color traits, with an emphasis on the implications that these mechanisms have for understanding the evolution of these traits and how pigmentation can constrain different aspects of avian biology.

## 2. Cell Biology of Bird Melanization: Avian Melanosomes

Although melanosomes and melanin granules have been extensively studied in mammals, avian feathers were a good model for the early electron microscopy studies of this subcellular organelle and melanogenesis. It appeared that the avian material offered especially favorable conditions for such studies in comparison to mammalian skin. A single barb or barbule washed in acetone, dried and mounted for examination shows a very thin layer of keratin that is relatively transparent to the electron beam, so that sample preparations were easy for such pioneer studies. Thus, comparison of the microscopic structure of black feathers from the Brown Leghorn male chicken, the brown ones of the female, and Rhode Island red fowl feathers suggested the existence of two types of melanosomes containing two different types of melanin [10].

The granules from the black breast feathers of the Brown Leghorn male consisted of dense ovoids about 1 μm long, with a smooth and sharply defined edge and zigzag longitudinal strands. Rhode Island Red feathers showed granules with different structure. They were discoidal, quite uniform in size, less organized, without internal strands and empty vacuoles. White feathers were quite devoid of granules. In sum, these studies highlighted that the change from black to red color was not merely caused by a change in the nature of the pigment-containing granule, but also by changes in the genesis and structure of the granule. These ultrastructure differences between subcellular organelles forming eumelanin and pheomelanin were observed in other avian models, such as melanocytes from regenerating feathers in the pink-eye fowl mutation [11,12].

The incorporation of tyrosinase (the key enzyme for melanogenesis) to premelanosomes was also investigated in regenerating fowl feathers by histochemical staining with dopa [13], showing that coated vesicles were active in the transport of tyrosinase to initiate melanin formation. However, the avian melanin granules were not a good model for further tyrosinase studies, as those granules were mature organelles where most melanin is already formed and tyrosinase is mostly inactive after the previous period of melanin formation in melanosomes at the follicular melanocytes.

Studies on fowl showed that two parallel processes take place in developing feathers: growth caused by keratin deposition, and deposition of melanin. Basal keratinocytes form a ring from which the rachis and barb ridges differentiate. Melanocytes, initially located in the dermal papillae below the keratinocyte ring migrate towards the barb and simultaneously transfer melanin granules to the barbs and barbules of the definitive feather. Similar to mammalian skin and hair, the process of melanin transfer includes the incorporation of single melanosomes or melanosome complexes to the cytoplasm of keratinocytes that are incorporated to the growing feathers. Further studies reported that the pattern of formation and transference of melanosomes in bird feathers showed some differences in comparison to mammalian hair [14], but the detailed mechanism is still not totally clarified.

Recently, it has been proposed that the transfer of melanin from mammalian melanocytes to keratinocytes is carried out by a coupled exocytosis of the “melanocore” of melanosomes, with a subsequent endocytosis by keratinocytes rather than a heterophagocytosis of the melanocyte dendrite tip or the release of melanosome-loaded vesicles [15]. By contrast, almost nothing is known about the transfer of melanins from follicular melanocytes to feathers. Lin *et al.* [16] have emphasized the importance of the avian feather as model for the cellular and molecular studies on the formation of pigment patterns. They found that melanocyte progenitors are distributed as a horizontal ring in the proximal feather follicle, sending melanocytes vertically up into the epithelial cylinder, which gradually emerges as feathers grow. However, how melanins are embedded in the growing keratinized substance of the feather vane might require some specific processes different of the mechanisms of transfer in the melano-epidermic unit.

During the last years a new aspect of interest in avian melanosomes has been originated from studies on fossil feathers because of the possibility of inferring the color of extinct birds and non-avian theropod dinosaurs. Although the term melanosome is widely used in these studies to refer to the fossilized structures, they actually deal with the morphology of fossil melanin granules. The term melanosome would indicate a functional organelle in a melanocyte instead of melanin granules that are pumped out of the organelles to be deposited in feathers. In any case, in these studies it has been assumed that rod shape granules contain eumelanin while spherical, smaller granules contain pheomelanin. Some of these studies (e.g., [17,18]) base this assumption on previous work on human hair melanin granules that used relatively aggressive treatments to extract melanins from the hair matrix [19]. As pheomelanin granules are less resistant to mechanical stress than eumelanin granules, such treatments might break pheomelanin granules making them appear spherical in contrast to unaltered eumelanin granules. Indeed, they noted that the shape of pheomelanin granules was more heterogeneous than that of eumelanin granules, so both spherical and rod shape granules of pheomelanin were actually observed [19]. Furthermore, a recent study using a slightly less aggressive extraction of melanins has reported rod shape granules from red, pheomelanin-based chicken feathers [20]. Therefore, granule morphology may not be a good predictor of the chemical composition of melanins that they contain, something that should be further explored.

Other studies on fossil feathers have established the average morphology of melanin granules in feathers of extant birds exhibiting different colors (black, brown, grey) to infer the color of fossil feathers on the basis of their morphology [21,22,23,24]. However, detailed studies on the color generated by the different chemical forms of melanins are lacking, so a limited variety of colors can be determined from the latter studies. As an additional cautionary note, there is an open discussion about the possibility that these fossilized granules are actually preserved bacteria [25]. In sum, more caution is needed when taking the predictive capacity of melanin granule morphology to make deductions on the integument coloration of extinct birds and dinosaurs.

## 3. Feather Melanin Isolation and Methods for Structural Determination

As mammalian hair, feathers contain two different types of melanin: (a) eumelanin giving a dark black, brown or grey appearances; and (b) pheomelanin, leading to a lighter yellowish to reddish appearances. In most cases, feather melanins are a mixture of eu- and phaeomelanin, although the relative amounts of each pigment differ with the avian species [26]. First isolation protocols involved the use of alkaline conditions to disrupt the hard keratin matrix and release the pigment, but alternative mild protease-treatments can be also used.

The most reliable method for melanin identification and quantification is high performance liquid chromatography (HPLC) analysis of chemical degraded samples [27], and later improvements [28,29]. Feathers are trimmed, washed with water and digested with acidic permanganate or hydriodic acid for eumelanin or pheomelanin analysis respectively. Resulting PTCA (pyrrole-2,3,5-tricarboxylic acid) and AHP (4-amino-3-hydroxyphenylalanine) are used as markers of eumelanin and pheomelanin, respectively, with conversion factors of 50 and 9 when applied to feathers [8,26,27,28]. Alternative degradation treatment has been later introduced for pheomelanin, based on the alkaline oxidation with H_2_O_2_. Under these conditions, quantitated markers are TTCA (thiazole-2,4,5-tricarboxylate) and TDCA (triazole-4,5-dicarboxylate) [29,30,31].

One of the main difficulties in the melanin analysis is the inability to study melanin structure in unaltered state. To solve this problem and to develop direct methods of melanin analysis, Liu *et al.* [32] have used synchrotron-based photoionization mass spectrometry (MS) to determine the composition of feather melanins. Roughly, it has been found that brown plumage is related to greater content of benzothiazole units, black plumage to dihydroxyindole oligomers and grey to benzothiazine units. GC/MS has also used for pheomelanin, as it can distinguish benzothiazines from benzothiazoles [33]. Electron paramagnetic resonance (EPR) and X-ray photoelectron spectroscopies have been also applied to explore the properties of eumelanin in silky fowl tissues [34]. Finally, the use of Raman spectroscopy as a simple, non-invasive technique to identify and quantify melanins in feathers and hairs has been recently proposed [35,36]. Raman spectroscopy gets different spectra for eumelanin and pheomelanin without the need of extracting the pigments, and is potentially applicable to any biological tissue.

## 4. Melanin Synthesis Pathway with Special Reference to Avian Melanin

Melanogenesis is not a unique and unaltered biosynthetic pathway, as animals, plants and microorganisms show some differences in the nature of precursors and the enzymatic machinery [9] but all pathways show common features: an initial phase consisting of an enzymatic-catalyzed oxidation of phenolic precursors to quinones, and a second phase consisting of unregulated polymerization of phenols and their related quinones.

**Figure 1:**
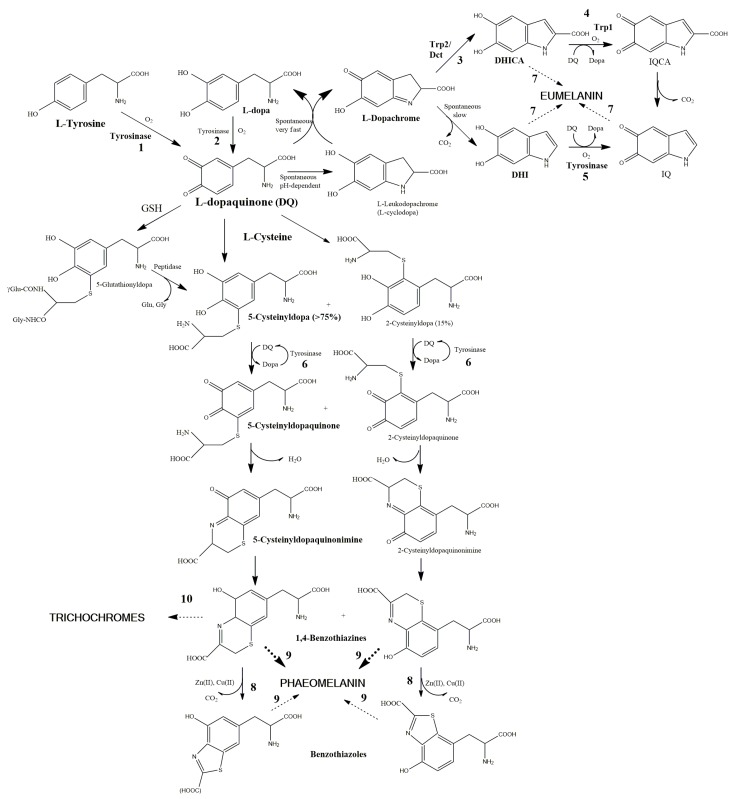
Melanogenesis pathway. l-Ty is oxidized to l-dopaquinone by tyrosinase. l-dopaquinone is a branch point to eumelanogenesis (internal cyclization to indole, the classical Raper–Mason pathway) or pheomelanogenesis (thiol addition on the aromatic ring). Other reactions lead to dark eumelanin (**upper** part) or yellowish to reddish pheomelanin (**lower** part). Enzymatic-catalyzed reactions are numbered 1–6, and metal ions l-catalyzed reactions are numbered 7–10, although traces of those metal ions can replace tyrosinase and Trps at reactions 2–6. Reaction 1 is the only key and rate-limiting step of the pathway. Mammalian and avian melanogenesis do not show differences concerning this essential step, but the availability of metal ions to catalyze rearrangements at the final steps may show differences among mammalian skin and avian feathers. See text for other details.

The most common and best-known route is called Raper–Mason pathway (Figure 1) [37,38]. Originally, this pathway was established as a route for the synthesis of dark eumelanin. Melanogenesis pathway was later extended [39,40] with a branch from l-dopaquinone leading to pheomelanin (called the Prota–Rorsman pheomelanogenesis branch in honor of these two pioneer researchers of pheomelanin chemistry). Pheomelanin is widely distributed among birds, which is related to the fact that this type of pigment was reported for first time in red feathers and hair [39,41].

In the initial step of melanogenesis, l-tyrosine is oxidized by oxygen in a tyrosinase-catalyzed reaction. Tyrosinase displays a complex mechanism of action [42] with two consecutive different activities: tyrosine hydroxylase and dopa oxidase. Both actions take place consecutively to generate l-dopaquinone (Figure 1). This *o*-quinone plays a pivotal role in animal melanogenesis [43] leading to eu- or pheomelanin depending on the chemical composition of the solution. l-dopaquinone is highly reactive, able to react with a number of chemical groups including amino, thiol and hydroxy groups. In the classical Raper–Mason pathway, l-dopaquinone undergoes a spontaneous intramolecular cyclization to be converted into l-leukodopachrome (l-cyclodopa) by a nucleophilic attack of its side chain amino group on position 6 of the aromatic ring. The rate of this reaction is highly dependent of pH, as the reactive species for the attack is the neutral amino group (–NH_2_), non-protonated. The pK of the amino group of l-dopa is approximately 8.7. Accordingly, eumelanogenesis is dependent of the pH of the media, being inhibited in acidic intramelanosomal media [44].

Following the eumelanogenesis pathway, l-leukodopachrome is an unstable indoline with strong reductant properties, so that it undergoes a spontaneous fast redox reaction with its precursor l-dopaquinone to regenerate half of l-dopa and to yield half l-dopachrome (Figure 1). l-dopachrome is a second pivotal intermediate in the melanogenesis pathway specific of eumelanogenesis [45,46,47]. l-dopachrome tends to evolve to dihydroxyindoles with a putative decarboxylation, so that two different intermediates can be formed, 5,6-dihydroxyindole (DHI) and –5,6-dihydroxyindole-2-carboxylic acid (DHICA). Actually, mixtures of both indoles appeared, but the ratio depends on several factors, such as pH, presence of metal ions and the action of a specific enzyme, dopachrome tautomerase (Trp2, tyrosinase-related protein 2) [45,48]. In mammals, the main factor is the activity of this enzyme, which has been well studied and characterized. In birds, this activity seems to be quite low and is has been rarely determined [49]. The presence of traces of Cu(II) or Zn(II) in follicular melanocytes could also favor the transformation of a portion of l-dopachrome into DHICA rather than into DHI.

In the final phase of the eumelanin pathway (Figure 1), both DHI and DHICA are putative substrates of tyrosinase or other enzyme involved in the eumelanin synthesis, tyrosinase-related protein (Trp1), to yield the corresponding 5,6-indolquinones [50]. The specificity of tyrosinase and/or Trp1 to the carboxylated or decarboxylated species shows differences between mouse and human [51], but to our knowledge there are no data about the specificity of avian tyrosinase and Trp1. Subsequent crosslinking reactions between oxidized forms (5,6-indolequinones) and reduced ones (5,6-dihydroxyindoles) generate the eumelanin pigment.

Returning to the pivotal point of the general melanogenesis pathway, l-dopaquinone proceeds to pheomelanin formation in the presence of l-cysteine or other thiol-containing compounds, such as the antioxidant GSH [40]. This is due to the much faster reaction of l-dopaquinone with thiol groups in comparison to the intramolecular cyclization leading to eumelanin [43,52]. Thus, in the presence of those agents, the thiol addition occurs, especially at acidic pH found inside pheomelanosomes. The addition of l-cysteine to l-dopaquinone can take place at different positions to yield a mixture of Cys-dopa derivatives, although the main isomer is 5-*S*-Cys-dopa [53,54] followed by 2-Cys-dopa (Figure 1, middle part) and other di-cysteinyl derivatives. If the thiol is GSH, 5-*S*-glutathionyl-dopa is predominantly formed, although a peptidase converts this derivative in 5-Cys-dopa by release of glutamate and glycine [40].

Cys-dopa isomers undergo oxidation to Cys-dopaquinones by mechanisms that are only partially known. Tyrosinase might barely catalyze these reactions, as available data indicate that mammalian tyrosinase shows very low affinity to Cys-dopas, making them very poor substrates [43,55]. There are no available data about affinity of avian tyrosinase to Cys-dopa. These oxidations have been extensively studied at Naples by Napolitano and d’Ìschia. However they approach biomimetic conditions at neutral pH with peroxidase plus hydrogen peroxide or inorganic oxidants, but no avian tyrosinase. Thus, it remains a low possibility that *in vivo* reactions in avian feathers could proceed under slightly different mechanisms than those described. Under these conditions, Cys-dopas are oxidized to Cys-dopaquinones through an intermediate dehydration to cyclic Cys-*o*-dopaquinonimines (Figure 1) and then to 1,4-benzothiazines (BTZ) [56,57]. Inside melanocytes, the mechanism for the oxidation of Cys-dopas to Cys-dopaquinones might be the redox reaction with l-dopaquinone that is reverted to l-dopa [43]. Thus, tyrosinase indirectly catalyses the oxidation as shown at Figure 1 although Cys-dopas are not direct substrates. 

The formation of carboxylated 2,3-dihydrobenzothiazines (DHBCA) before 1,4-BTZ has also been suggested [58], but a fraction of 1,4-BTZ evolves to related heterocycles, such as benzothiazoles, as significant proportions of these derivatives are detected by degradation of natural and synthetic pheomelanins [59]. Other more conjugated compounds, such as benzothiazolylthiazinodihydroisoquinolines, have been identified during the final polymerization reactions of pheomelanogenesis. These units have not shown at Figure 1 to avoid extra complexity. In some of these oxidative rearrangements, traces of Zn(II) [60,61] and other ions [62] seem to regulate final reactions (Figure 1). The transformation of benzothiazines to benzothiazoles is a complex organic rearrangement poorly understood that implies decarboxylation of the heterocycle ring [57]. The mixtures of those units yield a pheomelanin polymer containing different proportions of them [53,63]. The chemical factors to control a ”clean” dimerization of benzothiazines leading to trichochromes against the formation of benzothiazoles and other species leading to pheomelanins remains unknown. Zn(II) ions seem to be involved in both processes [64]. Although conditions leading to trichochromes or ill-defined pheomelanin should be clarified, there is no doubt that these variations account for the different colors in yellowish to red avian feathers.

## 5. Types and Building Blocks in Bird Melanin

Eumelanin and pheomelanin are present in mammals and birds, and there are no clear differences between both types of animals. However, due to the high diversity in feather coloration, it is likely that pheomelanins are more widely distributed, and maybe more chemically diverse, among birds. Different models help to understand the structural properties of these polymers [9,29,31,43]. Briefly, the main building block of eumelanin is DHI. Possible positions for polymerization in this unit are 2, 3, 4 and 7 [9,29]. Covalent bonds 4→7 are the most abundant cross-link among units, but positions 2 and 3 are also possible. This would give place to a branched and relatively flat polymer. On the other hand, as some of these units are carboxylated (DHICA units), position 2 is blocked, and position 3 is mostly inactivated by the electron withdrawing effect. Thus, DHICA tends to link through linear 4→7 bonds. The ratio DHI/DHICA in a eumelanin molecule depends on the dopachrome tautomerase activity (Dct) activity and/or the presence of metal ions [46,48]. Units of l-dopa, 5,6-indole quinones or carboxylated pyrrolic units could also be incorporated in low proportion to the polymer during its formation [43].

PTCA (Pyrole-2,3,5-tri carboxylic acid) and PDCA (Pyrrole-2,3-di) were established as the classical markers of DHICA and DHI units in eumelanin after chemical degradation of eumelanin. More recently, PTeCA (pyrrole-2,3,4,5-tetracarboxylic acid) and isoPTCA (pyrrole-2,3,4-tricarboxylic acid) have also been detected in very old melanin ink sacs [31,43], suggesting extra cross-linking of DHI units during the aging of the eumelanin polymer. PTeCA/PTCA ratio has been suggested as a good indicator of eumelanin aging. According to that aging process, it has been proposed that the size of the mature eumelanin molecules is large due to the complex mixture of different dihydroxyindole intermediates and the possible cross-linking among those units. Recently-formed pigment molecules, especially from DHICA, can be formed by small oligomers, such as dimers, trimers and tetramers [29,30].

On the other hand, it has proposed that eumelanin is comprised at least partially of non-covalent supramolecular aggregates formed by self-aggregation of l-dopa [65]. The monomers would be linked together by a combination of hydrogen bonds, π–π stacking, and ionic bonds. This report suggests that unmodified l-dopa may be part of the building blocks for eumelanin, but it needs confirmation, as it implies that tyrosinase would not be the rate-limiting step of the pathway, as l-dopa would be incorporated to the melanin polymer without oxidation.

Pheomelanin is more treatable than eumelanin, as it is more soluble than eumelanin due to its lower degree of conjugation [66]. Most of this pigment can be dissolved in alkali or acid media and it consists of oligomers formed by sulfur-containing units, mostly benzothiazine and benzothiazole (see Figure 1). More conjugated complex units such as benzothiazolylthiazinodihydroisoquinoline rings have been proposed in the pheomelanin structure [61].

**Figure 2:**
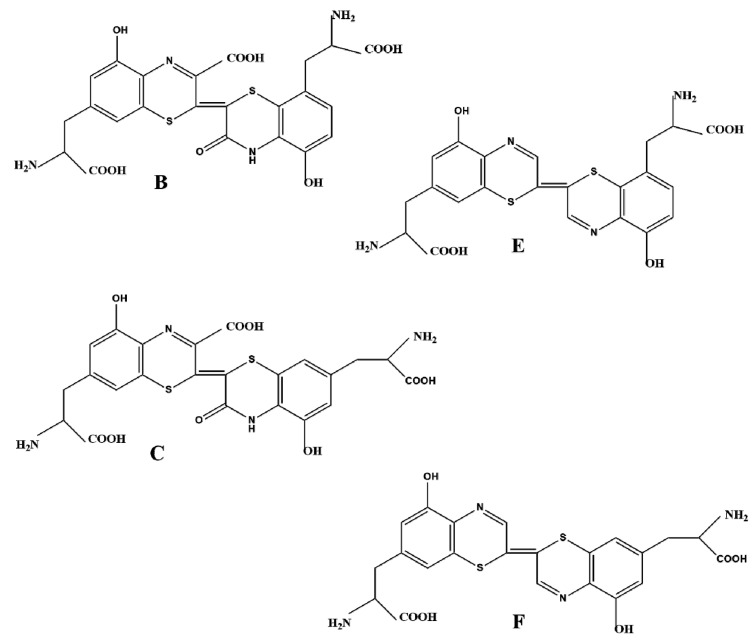
Trichochrome structure. These pigments are a type of phaeomelanin formed after benzothiazine dimerization. The four main structures, **B**, **C**, **E** and **F**, are shown. They have been isolated from red hair and red hen feathers.

In addition to eu- and pheomelanins, some reviews on avian pigments [67] consider two extra minor types of avian melanins, termed trichosiderins and erythromelanins. The term trichosiderin is equivalent to trichochrome, which is the recommended term [41]. Trichochromes are dimers of benzothiazines, so that they should be considered a particular type of soluble pheomelanins. The two most abundant trichochromes isolated from red chicken feathers are B and C, that contain a carboxyl group in the 3-position of the first benzothiazine and a keto group in the 3-position of the other unit. The isomer B is formed by the conjugation of 5-cysdopa with 2-cysdopa, whereas trichochrome C is formed by conjugation of two units of 5-cysdopa. When trichochromes B and C are heated in acid media, a decarboxylation takes place to yield decarboxytrichochromes, which leads to a change in color from yellow to pinky red [41]. Similar natural trichochromes E and F, devoid of carboxyl and keto groups, were be also isolated by these authors from red feathers (Rhode Island hen), although in lower amounts than trichochromes B and C (Figure 2). All these trichochromes were isolated by thin layer chromatography [41], from feathers, red hair and melanoma extracts, although red feathers showed greater number of isomers.

Concerning erythromelanins, they were hypothesized to justify the wide range of reddish color found in some bird species, but in fact, they have never been chemically characterized. Currently their existence with a different structure is doubtful Melanin granules composed by pheomelanin or mixtures of pheo- and eumelanin with other non-melanin pigments or morphological structures are enough to explain all structural colors. For a detailed anatomical description of the types of iridescent and non-irisdescent arrays of keratin, melanin and air in avian feather barbules, see [68].

In addition, during melanin synthesis, some of the o-quinonic precursor units can be conjugated not only with free cysteine or GSH, but also with thiols of side chains of a number of proteins, forming melanoproteins [69]. Melanoproteins were identified for the first time in melanin from cuttlefish ink, but were also described in other sources [31]. In animals with Dct activity, the incorporation of indole to proteins could be much lower, as this enzyme forms DHICA instead of DHI and it thus decreases the incorporation to the protein chain [70]. As keratins are rich in cysteine, the possibility of l-dopaquinone addition at the side chains of cysteinyl residues is a reasonable possibility that deserves being explored although most of Cys residues are involved in disulfide bridges to maintain the structural stability of keratin fibers.

## 6. Environmental and Genetic Control on Avian Melanogenesis

Melanin synthesis is environmentally and genetically controlled since the pathway involves precursors and traces of some metal ions mainly in the pheomelanogenesis pathway, but also enzymes and specific hormones. Melanin precursors (*i.e.*, tyrosine, its precursor phenylalanine, and cysteine) and metal ions are acquired with the diet, thus making diet quality a potential influence on melanin pigmentation [71,72]. Furthermore, the fact that cysteine and GSH, which is the most important intracellular antioxidant, play a key role in the melanogenesis pathway makes the synthesis of melanin open to environmental factors that generate oxidative stress, as GSH is depleted under exposure to such factors [73,74].

The genetic control of avian melanogenesis is exerted by genes coding for specific enzymes involved in melanin synthesis and other important regulatory and structural proteins also needed for the process. In animals, there are more than 120 genes involved in coat color determination [75], and more than 50 of those have been identified in birds [76]. Most of these genes code for melanogenic hormones, receptors, factors for melanoblasts migration and melanocyte differentiation, structural proteins and membrane transporters. Thus, the genetic control of melanogenesis implies hormones, termed melanocortins, all derived from processing a common precursor encoded by the pro-opiomelanocortin gene. The melanocortin system consisted of α-, β- and γ-melanocyte-stimulating hormones (MSH) and adrenocorticotropin (ACTH). Other important genes encode for five melanocortin receptors (MCRs) and some important regulators of the hormone-receptor interactions, as the agouti (ASIP) and agouti-related (AGRP) proteins [77,78].

**Figure 3:**
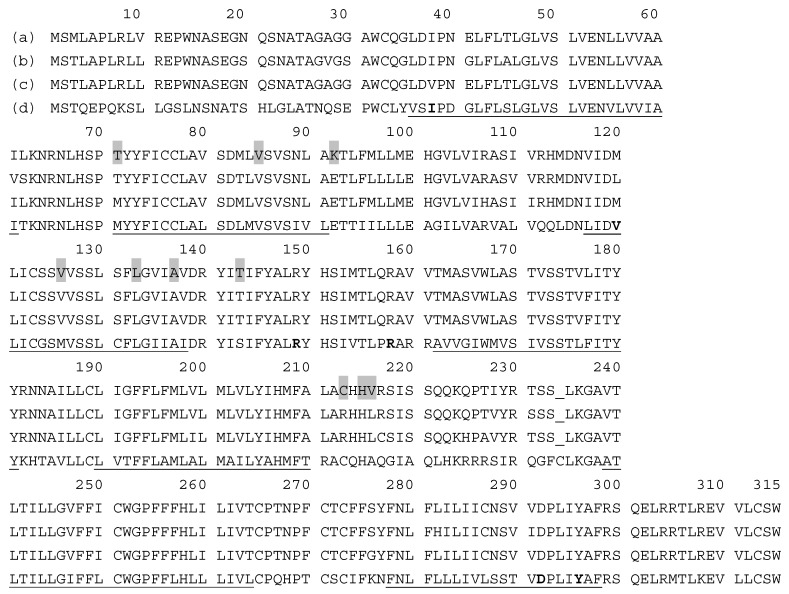

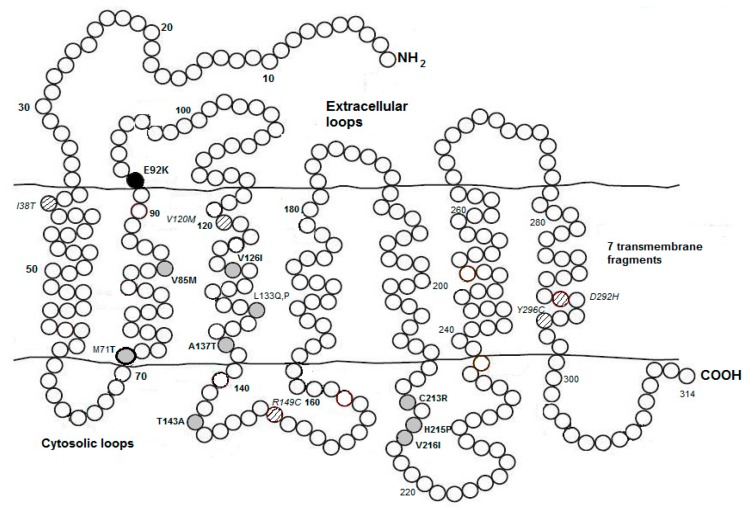
Melanocortin receptor 1 (MC1R) sequences and topological scheme of selected species: (**a**) Chicken (Red Jungle fowl, *Gallus gallus*) Acc. No. P55167-1; (**b**) Barn owl (*Tyto alba*), A0A0E3Z8U6-1; (**c**) Duck (*Anas platyrhynchos*) U3HZ58-1; and (**d**) Mouse (*Mus musculus*), Q01727-1. Sequences from www.expasy.org database. Transmembrane regions are underlined in the mouse sequence (line 4). Gray background residues (line 1 in sequences alignment and gray circles in scheme) are positions described in avian species determining the type and amount of melanin formed (see text for details). E92K replacement (black circle) causes constitutively-activated MC1R, so that dark melanin is formed even in the absence of hormone signal, MSH. Bold residues (line 4, in alignment, hatched circles in scheme) are mouse positions equivalent to those related to the RHC phenotype (red hair color) in the ortholog human protein. Surprisingly, most of these residues have not yet been reported as important in determining bird color plumage.

Regarding receptors, melanocortin receptor 1 (MC1R) is the most important one in all animals. MC1R up-regulates the expression of tyrosinase and two enzymes specific of the eumelanogenesis pathway: Tyrp1 and Tyrp2 (Dct). The activity of this receptor switches-on/off the synthesis of black/brown eumelanin or reddish/yellowish pheomelanin, as first described in chicken [79]. When this receptor is active and functional, dark eumelanins are formed in response to melanocortins, but a number of amino acid substitutions lead to variations in plumage coloration [78]. The best characterized replacement in the avian *MC1R* gene is at position 92. Glu92Lys substitution locks MC1R in an active state even in the absence of the hormone, at least in the chicken and tropical birds such as the bananaquit (*Coereba flaveola*) [80]. On the other hand, other amino acid replacements favor pheomelanogenesis, such as Val85Met in domestic rock pigeons (*Columbia livia*) [81] or the conservative substitution Val126Ile in barn owls (*Tyto alba*) [82]. Several missense mutations in this gene have been associated to almost complete white plumage color morphs rather than to eumelanin-based coloration in gyrfalcons (*Falco rusticolus*) [83], although the involvement of other loci different of *MC1R* cannot be ruled out. The association between *MC1R* polymorphisms and plumage coloration has been studied in 13 Spanish chicken breeds carrying six different alleles in the *MC1R* locus [84]. These chickens derived from domestication of a single ancestor, the red jungle fowl (*Gallus gallus*) and they show a variety of plumage colors. The following substitutions have been reported: Met71Thr, Glu92Lys, Val126Ile, Leu133GlnPro, Ala137Thr, Thr143Ala, Cys213Arg, His215Pro, and Val216Ile. Overall, it seems that Lys92 leads to a constitutive activated receptor leading to eumelanin production even in the absence of melanocortins, although it seems to be necessary but not sufficient to express the extended black phenotype, as the Cys213Arg change may be the cause of the loss of function to produce eumelanin, and the Ala137Thr one may be a candidate to attenuate Glu92Lys effects. Figure 3 displays the alignment of sequences of three typical avian receptors (chicken, owl and duck) in comparison with the MC1R mouse protein as a mammalian reference. A scheme of the MC1R inserted in the melanocyte membrane is also shown.

MC1R also up-regulates the main structural intramelanosomal protein, Pmel. This protein was described in birds and mammals, and its truncation gives place to the *silver* mutation in mice [85] has been confused for years since the protein was named differently Pmel17, gp100, gp95, gp85, ME20, RPE1, SILV, MMP115 in chicken [86], and finally simply Pmel, pre-melanosomal protein [87]. Mutations in the *Pmel* gene cause hypopigmentation in chicken, being responsible for the dominant white, dun and smoky alleles that inhibit the expression of black eumelanin [88] Feather-pecking in chickens, a behavior that can lead to the death of victim birds, is more frequently observed when the plumage color of the victim is associated to the expression of a wild recessive allele at the *Pmel* gene [89]. Thus, *Pmel* is important to improve welfare conditions in poultry. Concerning the enzymatic regulation of melanogenesis, tyrosinase is the most important enzyme, as it catalyzes the rate-limiting steps in the pathway. Chicken tyrosinase seems to be more acidic than the corresponding mammalian enzyme [90]. In that way, chicken and probably other avian tyrosinases seem to be adapted to act in mild acidic conditions in comparison to mammalian tyrosinase. These acidic conditions might favor pheomelanin conditions in comparison to eumelanin formation, taking into account the acidic conditions existing within melanosomes [44] and that cysteine addition is favored to l-dopaquinone cyclization under those conditions. *Trp1* and *Trp2* were cloned in birds [91,92], but their level of activity in follicular avian feather have been rarely determined. However, their expression has been correlated with darker pigmentation in a number of bird species, especially Trp1, which is involved in the final phase of eumelanogenesis by exerting DHICA oxidase activity [50]. Thus, both tyrosinase and Tyrp1 activity levels account for melanin-based plumage color variation in chickens, ducks, Korean quails, pigeons and geese [93,94,95,96]. The role of avian Trp2 has not been studied in depth because the Dct activity is not essential for eumelanogenesis, but recently some SNPs in the corresponding *Trp2* gene have been associated with plumage color variation in Korean native ducks [97]. In addition to the role of pH, other essential requirement for pheomelanin synthesis is the availability of cysteine or GSH. Certainly, protein transporters for H^+^, tyrosine and cysteine/GSH are needed for melanogenesis. Concerning protons, the *p* protein (also named pink-eyed dilution protein) seems to act as a proton-dependent membrane transporter for tyrosine. Mutations in this protein are related to oculocutaneous albinism type 2 (OCA2). However, this protein has been poorly studied in the avian melanosome.

The Slc7a11 gene encodes a membrane cystine/glutamate exchanger whose inactivation reduces pheomelanin production in *sut* mice [75], suggesting a role in pheomelanin formation by regulating the transport of cysteine inside melanocytes. This protein is also critical for the normal proliferation of melanocytes, GSH production and cell protection against oxidative stress. In turn, a similar cystine/H^+^ symporter named cystinosin has been described in lysosomes and melanosomes [98]. Mutations in cystinosin produce a decrease in eumelanin and increase in pheomelanin formation in mammalian hair. Both transporters are therefore involved in the synthesis of pheomelanin but this role has not been investigated in birds.

There are other solute carriers involved in melanization, such as the potassium-dependent sodium–calcium ion exchanger Slc24a5, whose dysfunction is responsible for human OCA6. Down-regulation of this cation exchanger reduces pigmentation in chicken [99]. On the other hand, Slc45a2, also known as MATP (membrane-associated transporter protein) in mammals, has an important role in vesicle sorting in melanocytes and in trafficking melanogenic enzymes. Its mutation causes OCA4 in humans [100] and melanin-based plumage color variation in chicken and Japanese quail. The mechanism of melanin regulation and the pattern of *Scl45a2* mutations in birds is complex and poorly understood, as some mutations cause complete absence of pigmentation while other cause a specific inhibition of pheomelanogenesis [101].

Lastly, hormones that do not participate in the melanogenesis system, especially sexual steroids, also influence melanin pigmentation. This is prominently known for androgens such as testosterone, which favor the expression of dark eumelanin-based plumage traits in birds [102,103]. The influence of testosterone on eumelanogenesis could be mediated by oxidative stress generated by this hormone, which in turn may deplete GSH levels, thus promoting eumelanin synthesis [104]. It must be noted here that female birds can differentially transfer testosterone to the egg yolk [105], which leads to another source of environmental influences on melanin synthesis. Trace metals, which affect melanin synthesis as mentioned above, can also be transferred to the egg yolk [106] This capacity of females to differentially transfer testosterone and trace metals to the egg yolk also implies that cross-fostering experiments, sometimes used to estimate the heritability of melanin-based traits in birds [107], do not completely remove potential confounding parental effects and thus probably tend to overestimate heritability. In sum, melanogenesis in birds is under a relatively complex genetic control, but it is also open to at least four lines of environmental influences (trace metals, melanin precursors, environmental oxidative stress and maternally transferred testosterone and trace metals) that should be considered to understand the expression of melanin-based traits.

## 7. Function and Evolution of Bird Melanins

### 7.1. Protection against UV Radiation

The ubiquitous nature of dark melanins (*i.e.*, eumelanin) in living organisms, where they can be found from bacteria to humans, is probably related to their capacity to protect cells from the damaging effects of UV radiation. It can be stated that this represents the primary function that provides organisms with the main adaptive benefits making melanins evolve. This property is conferred by a high refractive index and broad absorption spanning across the UV-visible range of the electromagnetic spectrum, thus avoiding that cells suffer from the pro-oxidant substances generated by the highly energetic UV radiation. As a consequence, the presence of melanins in the integument is essential for the development of life on earth, melanization being considered the main physiological response of animals and microorganisms against the damaging effects of UV [108]. While this generalization for the protective function of melanins is strongly founded, empirical evidence for it rarely comes from organisms other than mammals, prominently humans.

Birds constitute an outstanding example of lack of evidence for the protective role of melanins against UV radiation, although it is a common assumption. Variation in melanization levels in birds has been mainly studied regarding plumage, but skin melanization, which might be most relevant for protection of the organism against UV radiation, remains unexplored in birds [109]. In this regard, it is worth mentioning the absence of knowledge about prevalence of melanoma in wild birds beyond descriptions of particular cases [110]. The diurnal and aerial habits of most birds probably make them the vertebrates most exposed to UV radiation, and investigating how frequent is melanoma in wild birds may provide interesting information on possible mechanisms to avoid the damaging effects of UV radiation.

### 7.2. Protection against Mechanical Damage

Melanins are polymers that increase the hardness of the biological tissues where they are embedded [111]. In contrast to the previous protective function against UV radiation, the capacity of melanins to increase the resistance of integumentary structures to mechanical damage has been particularly well illustrated in birds. Thus, it has experimentally been shown that feather and bill melanization makes increase the hardness of these structures [112], as well as their resistance against abrasion caused by airborne particles [113]. It is unclear, however, if different chemical forms of melanins differ in their mechanical protective capacity, although the poor integrity of melanin granules from human hair [19] leads to expect that pheomelanin-based feathers are more fragile than eumelanin-based ones. However, the only study so far investigating this issue in birds concluded that eumelanin and pheomelanin do not differ in their capacity to increase plumage strength, although chemical analyses of melanins were not conducted and composition was inferred from the color of feathers [114].

Melanins can also protect feathers from the damaging effects of feather-degrading bacteria [115]. In fact, skin melanization has been proposed to be a primitive component of innate immune defense system [116] but it has also been suggested that melanins may serve as a substrate for some species of bacteria [117]. The latter work has not been replicated in detail [118], but it would be interesting to consider it before accepting as a fact that melanins protect against any feather-degrading bacteria. Similarly, there is no firm evidence that melanized feathers protect from feather-degrading lice, as while some authors concluded that the degree of damage by these organisms to feathers is not related to melanization levels [119], the method for lice quantification has recently been criticized [120]. Again, differential protective roles of different chemical forms of melanins have not been evaluated for feather-degrading lice.

### 7.3. Thermoregulation

The wide absorbance spectrum of melanins in the UV-visible range makes that these pigments protect cells from the damaging effects of energetic UV radiation, but this also implies that melanized biological structures usually increase their temperature more easily than non-melanized, lighter structures. For animals, this represents an advantage when increasing body temperature is necessary, but is also a physiological cost when thermal limits are exceeded, especially in ectotherms [121]. Birds, like mammals, maintain a relatively constant physiological temperature, which makes that the thermal function of melanins has been less investigated in these organisms. Indeed, the role of plumage melanization in covering the thermoregulatory needs of birds is unclear (reviewed in [109]). However, maintaining a constant body temperature may represent a significant physiological cost for birds when exposed to low ambient temperatures [122], and in such circumstances the ability of black, melanized plumage patches to rapidly absorb radiant energy may be adaptive. This may in fact favor the evolution of melanin-based traits characteristic of bird subspecies inhabiting cool environments [123]. However, this capacity of dark melanin-based colors to absorb radiant energy may actually represent a physiological cost under high ambient temperatures, which may have led to the evolution of compensatory mechanisms such as vascularized parts of bare skin that dissipate heat and whose colors caused by blood flow interestingly may have secondarily evolved as intraspecific signals of dominance [124,125]. It will be interesting to consider this potential physiological cost in a context of global warming, which may create differences in the vulnerability of species to extinctions regarding their genetic basis of melanin synthesis. Differential thermoregulatory properties of different chemical forms of melanins have not been explicitly evaluated in birds, but [126] have recently reported larger and denser plumulaceous parts of dorsal feathers in the grey morph (which mainly produces eumelanin) than in the brown morph (which mainly produces pheomelanin) in tawny owls *Strix aluco*, suggesting a superior insulative capacity in the former. The chemical form of melanin being produced might not be responsible for these structural differences in the feathers of both color morphs, but the authors suggest that these differences may explain the lower survival of brown, pheomelanic tawny owls during cold winters [127]. In our opinion, however, the fact that those structural differences between color morphs only appear in dorsal feathers, but not in ventral feathers, may suggest that the low survival experienced by pheomelanic tawny owls during adverse winters is actually related to the chemical form of melanin. In particular, it may be related to the cost represented by the consumption of cysteine/GSH during pheomelanin synthesis under thermal stress, which generates oxidative stress and reduces GSH levels [128].

### 7.4. Signaling

As in mammals, the range of colors conferred by eumelanin and pheomelanin to different parts of the body has favored the evolution in birds of an extraordinary diversity of plumage colorations and patterns as visual signals involved in interspecific communication by conferring crypsis to avoid either being predated [129] or being detected by prey [109]. Such diversity of colorations has also made that many melanin-based color traits in birds allow transferring information about attributes of the signal bearers to conspecifics. The information that melanin-based color traits can transfer can be related to the physical and chemical properties of melanins and to the processes underlying the chemistry of melanogenesis. Thus, the optical properties of melanins favor that melanin-based traits secondarily evolve as visual signals.

Most biological signals evolve because they are honest, *i.e.*, influence positively the receivers of the signals [130]. In animals, signal honesty can be achieved by either trade-off relationships between costs and benefits of signaling (*i.e.*, handicaps) or by signal design (*i.e.*, amplifiers and indices) [131]. Melanin-based color traits in birds often act as honest signals of quality, as signalers with larger or more intense color patches are perceived by conspecifics as bearers of a superior underlying genotypic quality and as a consequence achieve higher fitness benefits than others displaying smaller or less intense color patches [132,133]. There are probably examples of the two mechanisms that allow the evolution of honest signals (*i.e.*, handicaps and signal designs) among melanin-based color traits in birds.

The handicap principle [134] establishes that only high-quality individuals can afford the costs derived from signal production. The fact that melanogenesis proceeds in two main branches, one in the absence of cysteine/GSH leading to eumelanin production and other in the presence of cysteine/GSH leading to pheomelanin production, is useful to understand these costs, which will be mediated by the environmental conditions of oxidative stress. This may actually agree with the observed temporal variation in female preference by male melanin-based plumage traits in birds [135], suggesting that melanin-based traits are excellent models to study environmental influences on sexual selection. Pheomelanin-based color traits will be particularly costly to produce under high stress levels, when cysteine/GSH is more necessary to combat oxidative stress and pheomelanin synthesis represents a consumption of this antioxidant resource [73,74]. Thus, it should be expected that high stress levels promote the evolution of pheomelanin-based signals, when these could only be produced by high-quality individuals. By contrast, low stress levels may promote the evolution of eumelanin-based color signals, as producing eumelanin under such circumstances may require getting rid of cysteine/GSH [73,74]. However, giving the existence of specific transporters of cysteine that can potentially regulate cysteine levels in and out of melanocytes, the latter possibility becomes less likely. This leads to speculate that pheomelanin-based color traits have a higher potential to evolve as honest signals of quality than eumelanin-based color traits, which may be in accordance with the apparent scarcity of bird species with plumage fully composed of colors typically conferred by pheomelanin (*i.e.*, chestnut, ferruginous) and the abundance of species fully colored by eumelanin (e.g., blackbird *Turdus merula*, common raven *Corvus corax*).

Regarding signals that achieve their honesty by design, the dark colors conferred by melanins, especially eumelanin, make that alternations of melanized (black) and unmelanized (white) plumage patches achieve the highest possible level of achromatic contrast and therefore generate very conspicuous traits. As a consequence, these unmelanized patches easily amplify properties of the bearers, for example their social status by improving the perception by others of feather damages produced by attacks received from conspecifics [136,137], or their capacity to perform ritualized movement displays [138]. These signals are honest because their design make impossible that the bearers’ attributes being transferred are faked. Thus, when melanin-based color traits evolve as signals, the size or intensity of color patches use is handicapped while patterns of melanized/unmelanized patches have a greater capacity to be amplifiers and indices.

Melanin-based color traits in birds have not only improved our understanding of the costs that maintain the honesty of signals, but have also provided insight into the evolution of signal honesty. Thus, for the black bib of the house sparrow *Passer domesticus*, whose size acts as a honest signal of quality, it has been proposed that the mechanism by which cysteine is consumed to produce pheomelanin in the bib feathers may be functional in high-quality birds but uncoupled in low-quality birds. As large signals are produced with low contents of pheomelanin in feathers (in the case of the black bib of house sparrows and other species as well [139], this would prevent low-quality birds from producing signals unproportionally large for their quality independently of the prevailing environmental conditions of oxidative stress [140]. Further explorations of this possibility may transform our view of biological communication, as signal honesty may be physiologically maintained without the need of production costs. Moreover, this also implies that the genes involved in pheomelanin synthesis may actually determine the basis of the concept of genotypic quality, at least for melanin-based color signals [140].

## 8. The Challenge of Pheomelanin Evolution

A particularly interesting issue in the study of melanins is the evolution of pheomelanin. This melanin form requires a consumption of cysteine/GSH and, as opposite to eumelanin, produces pro-oxidant species under exposure to UV radiation and probably also under exposure to ionizing radiation [141]. Therefore, why has this pigment evolved? This unsolved issue has led some authors to consider pheomelanin an accident of nature [142]. Adaptive benefits might be the response to this question, and studies with birds have been pioneering in exploring the adaptive benefits that pheomelanin may provide. Indeed, the consumption of cysteine during pheomelanogenesis may be adaptive in certain circumstances in which this amino acid is in excess and potentially toxic [143]. Studies on several species of birds have shown that producing pheomelanin can be beneficial and increase survival if there is no evidence of particularly high levels of environmental oxidative stress [144], but is also involved in physiological trade-offs as shown by negative relationships between the extent of plumage colored by pheomelanin and brain size [145], capacity to resist the effects of ionizing radiation [141], physiological stress caused by corticosterone [146], and survival during adverse environmental conditions [127]. The evolution of pheomelanin may therefore respond to the benefits conferred by the synthesis of this pigment by avoiding excess cysteine under low levels of environmental stress, representing a physiological cost under high levels of environmental stress. A promising avenue for future research will be to identify particular environmental conditions that the production of pheomelanin helps to cope with, in birds and other animals including humans.
